# Predictors for Home Discharge and Need for Medical Care at Home in Paediatric ICU Patients: A Single‐Centre Retrospective Study

**DOI:** 10.1111/nicc.70404

**Published:** 2026-02-23

**Authors:** Misuzu Oyagi, Shigeki Bamba, Kohei Takashima, Yasuhiko Imashuku, Hirochika Ryuno, Naoto Shiomi, Hirotoshi Kitagawa, Masahito Hitosugi

**Affiliations:** ^1^ Department of Fundamental Nursing Shiga University of Medical Science Otsu Japan; ^2^ Department of Anesthesiology Shiga University of Medical Science Otsu Japan; ^3^ Department of Pediatrics Shiga University of Medical Science Otsu Japan; ^4^ Division of Data Science for Clinical Nursing Shiga University of Medical Science Otsu Japan; ^5^ Department of Critical and Intensive Care Medicine Shiga University of Medical Science Otsu Japan; ^6^ Division of Legal Medicine Shiga University of Medical Science Otsu Japan

**Keywords:** children under medical care, medically complex, technology‐dependent

## Abstract

**Background:**

The number of children with medical complexity at home has been increasing. To facilitate a smooth transition to home care for these children after discharge from the intensive care unit (ICU), early nursing interventions and family training are essential.

**Aim:**

This study examined factors that predict home discharge and the need for medical care at home using routine clinical information at the time of ICU admission.

**Study Design:**

This study included paediatric patients under the age of 16 who were admitted to the ICU. Clinical data were extracted from medical records. Univariate and multivariate analyses were conducted using logistic regression to identify relevant factors. Receiver operating characteristic (ROC) analysis was performed to determine the cut‐off values.

**Results:**

One hundred and thirty‐one patients were included, of whom 82 patients were discharged home. Multivariate analysis using logistic regression identified the following independent predictors of home discharge: postoperative admission (*p* = 0.024), lower heart rate at admission (*p* = 0.016), absence of cardiopulmonary arrest immediately before ICU admission (CPA) (*p* = 0.012) and no mechanical ventilation within the first hour of ICU admission (*p* = 0.044). ROC analysis for heart rate at admission yielded a cut‐off value of 131 bpm (*p* = 0.039). A total of 62 patients did not require home medical care. Univariate logistic regression analysis identified length of ICU stay as a significant factor associated with the need for home medical care (*p* = 0.045).

**Conclusions:**

In paediatric ICU patients, postoperative admission, lower heart rate at admission, absence of pre‐admission CPA and no mechanical ventilation predicted successful home discharge. The need for home medical care was significantly associated with length of ICU stay.

**Relevance to Clinical Practice:**

Early and appropriate nursing interventions can be considered for paediatric ICU patients and their families, leading to the provision of high‐quality nursing care.

## Impact Statement

- What is known about this topic
  ○ The number of children with medical complexity (CMC) living at home has been increasing; many have prior intensive care unit (ICU) stays, underscoring the need for early family training to support transition to home care.
  ○ Existing paediatric severity scores (e.g., Paediatric Index of Mortality 2/3 [PIM2/PIM3]) are mainly designed to predict mortality or length of stay and have not examined admission‐time predictors of home discharge or need for home medical care.
- What this paper adds
  ○ Using routinely available admission data, this study identifies independent predictors of home discharge: postoperative admission, lower heart rate at admission, absence of cardiopulmonary arrest immediately before ICU admission and no mechanical ventilation within the first hour.
  ○ It provides a practical threshold—heart rate 131 bpm at ICU admission—associated with the likelihood of home discharge.
  ○ In a pre‐specified secondary analysis, longer ICU length of stay was associated with the subsequent need for home medical care (cut‐off ≈12 days), informing discharge planning and early family education.

## 1 Introduction

A ‘child with medical complexity (CMC)’ refers to a child who, following a prolonged hospitalisation in an intensive care setting such as a Neonatal Intensive Care Unit (NICU) or a Paediatric Intensive Care Unit (PICU), continues to require the use of medical devices such as a ventilator or a gastrostomy tube. These children necessitate daily medical interventions, including suctioning of secretions and enteral feeding [1]. The number of CMC at home has been increasing annually due to advances in medical technology. According to the Ministry of Health, Labour and Welfare of Japan, the estimated number was about 10 000 in 2005, but it had doubled to approximately 20 000 by 2021 [2]. Furthermore, a survey of CMC revealed that 58.2% of those receiving home care had experienced admission to an intensive care unit (ICU) [3]. To promote a seamless transition to home care for CMC, it is necessary for healthcare professionals to provide families with training and support for transitioning to home‐based care from the early stage of ICU admission.

In the evaluation of outcome predictions for ICU patients, correlations have been demonstrated with severity scoring systems such as the Paediatric Index of Mortality 2 (PIM2) and the updated PIM3 [4, 5, 6]. These evaluation indices are employed in studies where outcomes include mortality, long‐term hospitalisation and the Paediatric Cerebral Performance Category (PCPC) scale [7, 8, 9], and they have been shown to be associated with factors such as cardiopulmonary arrest (CPA), the use of mechanical ventilation, the use of inotropic drugs, the length of stay in the PICU, age under 2 years, PICU readmission and severity at the time of admission [10, 11, 12, 13, 14, 15, 16]. However, there has been no examination of outcomes that consider the possibility of discharge to home or the need for medical care at home.

Even when a child survives after discharge from the ICU, the nursing interventions differ between children who require medical care at home and those who do not. Therefore, it is crucial to initiate nursing interventions and family training from the early phase of ICU admission. To provide better nursing care, there is a need for indicators that can predict the possibility of discharge to home and the need for medical care at home.

## 2 Aim

The purpose of this study was to examine factors that can predict the possibility of home discharge and the need for medical care at home using routine clinical information at the time of ICU admission.

## 3 Methods

### 3.1 Participants

This study included paediatric patients under the age of 16 who were admitted to a university hospital in Shiga Prefecture, Japan between 1 January 2018, and 31 December 2022, and who could be followed for at least 6 months after discharge. Our hospital does not have a stand‐alone PICU; instead, paediatric patients requiring intensive care are admitted to the general ICU, which also cares for adult patients.

Inclusion criteria:

1. Age between 0 and < 16 years at the time of ICU admission.
2. Admission to the ICU for treatment purposes.
3. For patients with multiple ICU admissions, only the first admission was included.

Exclusion criteria:

1. Admission to the ICU for procedural purposes only (e.g., dialysis, central venous catheter insertion).
2. Patients whose guardians requested to opt out from the use of clinical information in this study.

After applying these criteria, 131 patients were screened. Among them, 13 patients were excluded: Eight for plasma exchange, two for continuous haemofiltration, one for peripherally inserted central catheter (PICC) insertion, one for central venous catheter (CVC) insertion and one patient who was initially admitted for plasma exchange but was discharged without undergoing the procedure.

### 3.2 Data Collection

1. Automatic extraction of medical information using the Philips Information Management System (PIMS) patient search function.
  The patient search function of the PIMS for critical and acute care patients was used to obtain the medical record identification number(s) (IDs) of paediatric patients under the age of 16 who were admitted to the ICU of our university hospital between 1 January 2018, and 31 December 2022. Furthermore, the medical information linked to those medical record IDs was automatically extracted and collected. The collected information included the patient's date of birth, age, gender, height and weight on admission, date and time of admission, date and time of discharge, diagnosis, surgical procedure and the route of ICU admission.
2. Review of medical records.
  For information that was unable to be automatically extracted using the PIMS patient search function and the linked data, the researchers directly reviewed the medical records to collect the necessary information. The collected data included the presence or absence of CPA before admission, the purpose of ICU admission, initial vital signs at ICU admission (heart rate, systolic blood pressure, diastolic blood pressure, respiratory rate, oxygen saturation, body temperature), the presence or absence of trauma, underlying medical conditions, catecholamine administration within the first hour of ICU admission, mechanical ventilation use within the first hour of ICU admission, the disease leading to ICU admission, the condition at discharge, medical care required at discharge and the destination after discharge. CPA immediately before ICU admission was defined as cardiopulmonary arrest that occurred immediately prior to ICU admission with successful resuscitation leading to ICU admission.

### 3.3 Statistical Analyses

The collected data were organised using Microsoft Excel for Mac 16.77 (Microsoft Corporation) and a database for analysis was constructed using JMP Pro Version 17.0.0 (SAS Institute). For comparisons between two groups, the chi‐square test was used to examine differences in proportions, and the Mann–Whitney U test was used to examine differences in medians. Logistic regression analysis was employed to identify independent factors influencing the likelihood of home discharge and the need for medical care. Hypothesis testing in logistic regression analyses was performed using likelihood ratio χ^2^ tests. Variables for multivariate analysis were screened through univariate analysis and appropriately selected based on existing evidence. The cut‐off values were calculated using Receiver operating characteristic (ROC) analysis. A significance level of 5% was set for all tests. Admission treatment variables (mechanical ventilation and catecholamine use) were defined as use within the first hour of ICU admission. The primary analyses focused on admission variables as predictors. In addition, ICU length of stay was also evaluated in a secondary analysis, as prolonged ICU hospitalisation may have practical implications for discharge planning and family education for home medical care, although it is not available at admission.

### 3.4 Ethical Considerations

This study was conducted with the approval of the Ethics Review Committee of Shiga University of Medical Science (approval number: R2023‐005, 14 April 2023). The study was registered in the University Hospital Medical Information Network (UMIN) Clinical Trials Registry (UMIN000050902). An opt‐out document was prepared, and consent to participate in the study was assumed based on its publication on the university website. The opt‐out document stated that it was possible to stop the use and provision of data in this study and clearly provided the opportunity to decline participation.

## 4 Results

**FIGURE 1:**
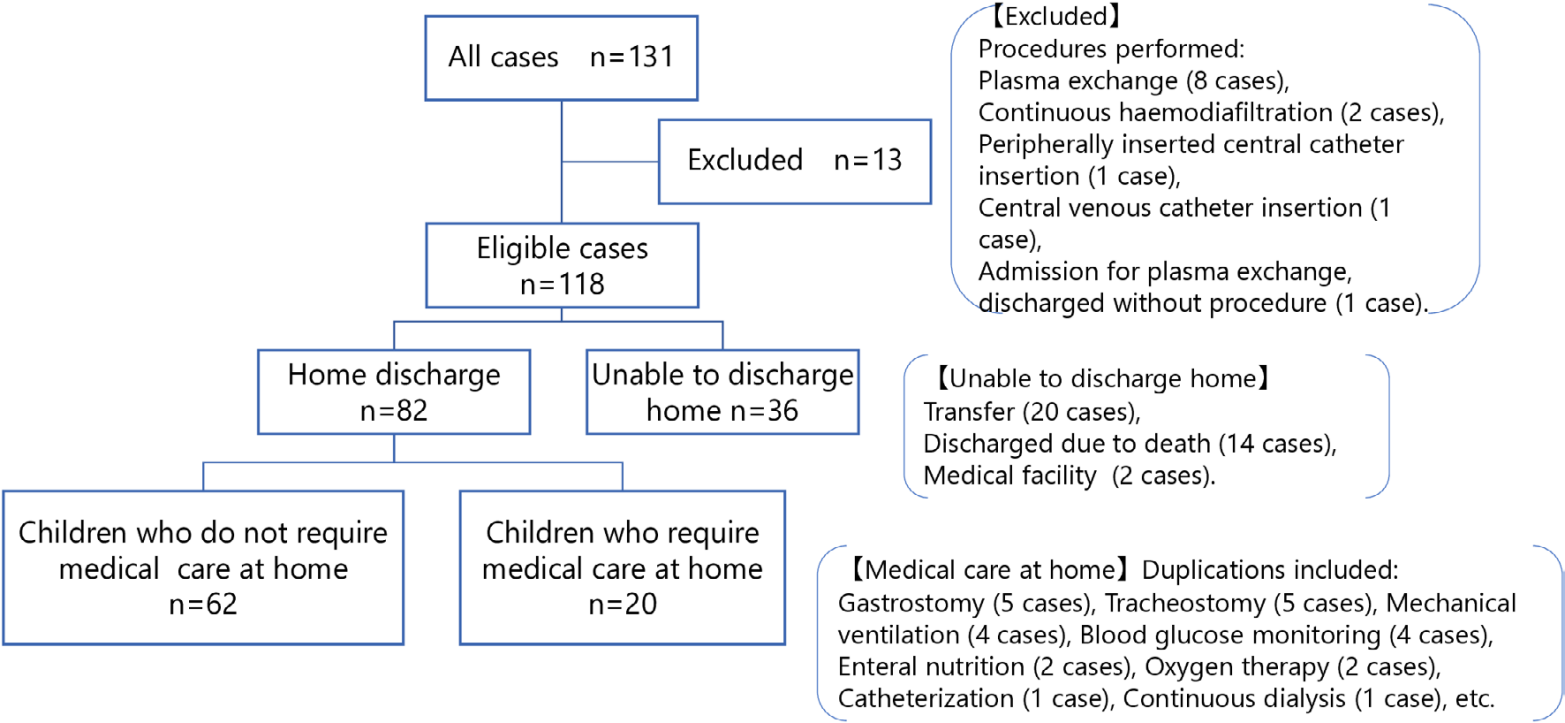
Flow chart of study participant.

The flow chart of study participants is shown in Figure 1. Of the 131 study participants, 13 patients whose purpose for ICU admission was a critical care procedure (e.g., 8 for plasma exchange, 2 for continuous haemofiltration) were excluded, leaving a final 118 patients for analysis. In this study, we first examined the feasibility of discharge to home, defined as discharge directly from the hospital to the patient's home without transfer to another facility, among paediatric patients admitted to the ICU. Subsequently, we investigated the need for medical care in patients who were deemed eligible for home discharge. Table 1 presents the basic characteristics of the entire patient cohort (*N* = 118) and those who were eligible for home discharge (*n* = 82).

**TABLE 1:** Basic characteristics of analysed subjects.

Patient background factors	All patients (*N* = 118)	Patients discharged home (*n* = 82)
Male/Female	78/40	54/28
Age (Years), median (IQR)	4.5 (1.0–8.8)	4.3 (1.2–9.4)
Height (cm), median (IQR)	99.3 (70.0–125.9)	101.0 (72.0–128.2)
Weight (kg), median (IQR)	15.1 (8.1–24.7)	15.8 (8.6–26.5)
Survival status (Survived/Deceased)	104/14	82/0
Discharge destination (Home/Transfer/Facility)	82/20/2	82/0/0
Length of ICU stay (Days), median (IQR)	3 (2–7)	3 (2–7)
Admission route (Transfer/ER/Operating room/Ward)	38/16/46/18	21/8/40/13
Heart rate (/min), median (IQR)	128 (113–154)	125 (110–145)
Systolic blood pressure (mmHg), median (IQR)	102 (84–115)	104 (88–115)
Diastolic blood pressure (mmHg), median (IQR)	62 (47–74)	63 (49–74)
Respiratory rate (/min), median (IQR)	25 (19–34)	24 (18–32)
Oxygen saturation (%), median (IQR)	99 (98–100)	100 (99–100)
Body temperature (°C), median (IQR)	37.0 (36.2–37.7)	37.1 (36.5–37.7)
Pre‐admission CPA^a^ (Yes/No)	13/105	2/80
Trauma (Yes/No)	7/111	3/79
Catecholamine use within the first hour of ICU admission (Yes/No)	20/98	8/74
Mechanical ventilation use within the first hour of ICU admission (Yes/No)	86/32	54/28

Abbreviations: CPA, cardiopulmonary arrest; ER, emergency room; ICU, intensive care unit; IQR, interquartile range.

^a^ Pre‐admission CPA was defined as cardiopulmonary arrest occurring immediately prior to ICU admission, with successful resuscitation leading to ICU admission.

**FIGURE 2:**
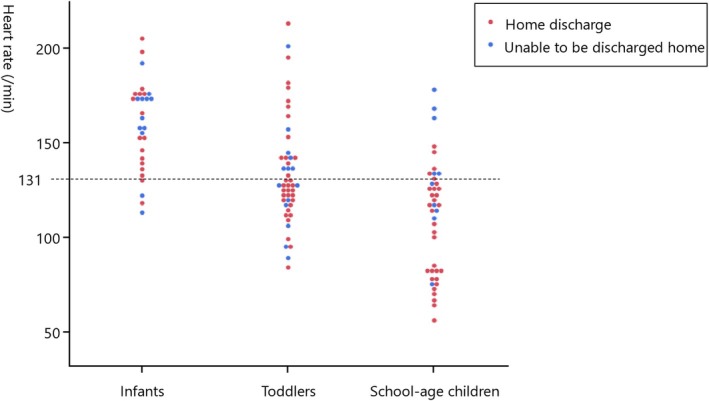
Association between heart rate and discharge to home (stratified by age). The heart rate at intensive care unit (ICU) admission was plotted by age group: Infants (under 1 year old), toddlers (1 to 6 years) and school‐age children (6 years and older). Cases with a home discharge outcome were marked in red, while those unable to be discharged home were marked in blue.

A logistic regression analysis was conducted to examine patient background factors associated with the feasibility of discharge to home among 118 patients (Table 2). In the univariate logistic regression analysis, the factors that were significantly associated with the likelihood of home discharge included postoperative admission (odds ratio (OR): 4.76, 95% confidence interval (CI): 1.89–13.78, likelihood ratio χ^2^ = 11.74, *p* < 0.001) and lower heart rate at admission (OR: 0.99, 95% CI: 0.98–0.99, likelihood ratio χ^2^ = 4.23, *p* = 0.039). In contrast, the presence of CPA immediately before ICU admission was associated with a lower likelihood of home discharge (OR: 0.06, 95% CI: 0.01–0.23, likelihood ratio χ^2^ = 18.74, *p* < 0.001), as were catecholamine administration within the first hour of ICU admission (OR: 0.22, 95% CI: 0.08–0.58, likelihood ratio χ^2^ = 9.14, *p* = 0.002) and mechanical ventilation use within the first hour of ICU admission (OR: 0.24, 95% CI: 0.07–0.68, likelihood ratio χ^2^ = 7.52, *p* = 0.006). All *p* values from logistic regression analyses were derived using likelihood ratio χ^2^ tests. ROC analysis was conducted for heart rate at admission, and the cut‐off value indicating the likelihood of home discharge was 131 bpm (sensitivity: 63.4%, specificity: 61.1%, area under the ROC curve [AUC] = 0.62, *p* = 0.039). Additionally, patients were categorised into three groups by age (infants under 1 year old, toddlers aged 1 to under 6 and school‐age children aged 6 and older), and the likelihood of home discharge was shown in Figure 2.

**TABLE 2:** Analysis of factors influencing feasibility of discharge to home.

Patients *N* = 118	Univariate OR (95% CI), *p*	Multivariate OR (95% CI), *p*
Male	0.96 (0.41–2.19), 0.931	—
Age (Years)	1.02 (0.94–1.12), 0.538	0.94 (0.83–1.06), 0.308
Height (cm)	1.01 (0.99–1.02), 0.295	—
Weight (kg)	1.02 (0.99–1.05), 0.320	—
Length of ICU Stay (Days)	0.96 (0.88–1.03), 0.242	—
**Admission route (Operating room/Transfer, ER, ward)**	**4.76 (1.89–13.78), < 0.001**	**3.21 (1.15–10.04), 0.024**
**Heart rate (/min)**	**0.99 (0.98–0.99), 0.039**	**0.98 (0.96–0.99), 0.016**
Systolic blood pressure (mmHg)	1.01 (0.99–1.02), 0.532	—
Diastolic blood pressure (mmHg)	1.00 (0.98–1.02), 0.864	—
Respiratory rate (/min)	0.96 (0.93–1.00), 0.076	—
Oxygen saturation (%)	1.02 (0.96–1.08), 0.503	—
Body temperature (°C)	1.22 (0.98–1.60), 0.080	—
**Pre–admission CPA** ^a^ **(Yes)**	**0.06 (0.01–0.23), < 0.001**	**0.13 (0.02–0.66), 0.012**
Trauma (Yes)	0.30 (0.06–1.45), 0.131	—
Catecholamine use within the first hour of ICU admission (Yes)	0.22 (0.08–0.58), 0.002	0.55 (0.15–2.11), 0.374
**Mechanical ventilation use within the first hour of ICU admission (Yes)**	**0.24 (0.07–0.68) 0.006**	**0.31 (0.08–0.97), 0.043**

*Note:* Bold values indicate statistical significance (*p* < 0.05).

Abbreviations: CPA, cardiopulmonary arrest; ER, emergency room; ICU, intensive care unit; OR, odds ratio.

^a^ Pre‐admission CPA was defined as cardiopulmonary arrest occurring immediately prior to ICU admission, with successful resuscitation leading to ICU admission.

Multivariate logistic regression analysis showed that the independent predictors for home discharge were postoperative admission (OR: 3.21, 95% CI: 1.15–10.04, likelihood ratio χ^2^ = 5.04, *p* = 0.024) and lower heart rate at admission (OR: 0.98, 95% CI: 0.96–0.99, likelihood ratio χ^2^ = 6.02, *p* = 0.016). In contrast, the presence of CPA immediately before ICU admission was associated with a significantly lower likelihood of home discharge (OR: 0.13, 95% CI: 0.02–0.66, likelihood ratio χ^2^ = 6.27, *p* = 0.012), as was mechanical ventilation use within the first hour of ICU admission (OR: 0.31, 95% CI: 0.08–0.97, likelihood ratio χ^2^ = 4.07, *p* = 0.043). These associations remained statistically significant even after adjusting for age in years. All *p* values from logistic regression analyses were derived using likelihood ratio χ^2^ tests.

Next, the results of logistic regression analysis examining the relationship between background and treatment information and the need for medical care at home are shown in Tables 3 and 4. In the univariate logistic regression analysis, the length of ICU stay (OR: 0.91, 95% CI: 0.82–0.99, likelihood ratio χ^2^ = 3.99, *p* = 0.045) was significantly associated with the need for medical care. Further ROC analysis was conducted, and the cut‐off value for length of ICU stay was 12 days (sensitivity: 96.7%, specificity: 25.0%, AUC = 0.60, *p* = 0.067). Among the 12 patients who stayed in the ICU for 12 days or more, seven required additional medical care. The types of medical care needed, with some overlap, included tracheostomy (5 patients), gastrostomy (3 patients) and mechanical ventilation (2 patients).

**TABLE 3:** Patient background factors and the necessity for medical care at home.

Patient background factors *n* = 82	Children who do not require medical care at home (*n* = 62)	Children who require medical care at home (*n* = 20)	*p*
Male/Female	41/21	13/7	0.926^a^
Age (Years), median (IQR)	5.0 (1.4–10.1)	2.3 (0.7–8.0)	0.256^b^
Height (cm), median (IQR)	102.1 (77.4–128.9)	87.7 (64.3–120.7)	0.241^b^
Weight (kg), median (IQR)	16.7 (9.7–28.4)	12.0 (6.8–21.5)	0.113^b^
Length of ICU stay (Days), median (IQR)	3 (2–6)	3 (2–11)	0.181^b^
Admission route (Transfer/ER/Operating room/Ward)	13/7/33/9	8/1/7/4	0.259^a^
Heart rate (/min), median (IQR)	125 (108–149)	126 (114–141)	0.766^b^
Systolic blood pressure (mmHg), median (IQR)	106 (87–118)	101 (92–107)	0.720^b^
Diastolic blood pressure (mmHg), median (IQR)	61 (48–74)	64 (51–76)	0.472^b^
Respiratory rate (/min), median (IQR)	23 (18–32)	24 (16–32)	0.995^b^
Oxygen saturation (%), median (IQR)	100 (98–100)	99 (99–100)	0.892^b^
Body temperature (°C), median (IQR)	37.1 (36.6–37.7)	36.8 (36.5–37.2)	0.251^b^
Pre‐admission CPA^c^ (Yes/No)	2/60	0/20	0.286^a^
Trauma (Yes/No)	3/59	0/20	0.190^a^
Catecholamine use within the first hour of ICU admission (Yes/No)	7/55	1/19	0.379^a^
Mechanical ventilation use within the first hour of ICU admission (Yes/No)	41/21	13/7	0.926^a^

Abbreviations: CPA, cardiopulmonary arrest; ER, emergency room; ICU, intensive care unit; IQR, interquartile range.

^a^ Chi‐squared test.

^b^ Mann–Whitney's U test.

^c^ Pre‐admission CPA was defined as cardiopulmonary arrest occurring immediately prior to ICU admission, with successful resuscitation leading to ICU admission.

**TABLE 4:** Analysis of factors influencing the need for medical care at home.

Patients *n* = 82	Univariate OR (95% CI), *p*
Male	1.05 (0.35–2.98), 0.926
Age (Years)	1.06 (0.95–1.19), 0.331
Height (cm)	1.01 (0.99–1.03), 0.242
Weight (kg)	1.04 (0.99–1.09), 0.073
**Length of ICU stay (Days)**	**0.91 (0.82–0.99), 0.045**
Admission route (Operating room/Transfer, ER, Ward)	2.11 (0.76–6.30), 0.153
Heart rate (/min)	0.99 (0.98–1.01), 0.761
Systolic blood pressure (mmHg)	1.01 (0.99–1.04), 0.270
Diastolic blood pressure (mmHg)	0.99 (0.96–1.02), 0.618
Respiratory rate (/min)	0.99 (0.94–1.04), 0.727
Oxygen saturation (%)	0.89 (0.62–1.02), 0.156
Body temperature (°C)	1.34 (0.73–2.49), 0.341
Pre‐admission CPA^a^ (Yes)	7.49e^−7^ (0–infinity), 0.286
Trauma (Yes)	2.71e^−7^ (0–infinity), 0.190
Catecholamine use within the first hour of ICU admission (Yes)	2.42 (0.39–46.71), 0.379
Mechanical ventilation use within the first hour of ICU admission (Yes)	1.05 (0.35–2.98), 0.926

*Note:* Bold values indicate statistical significance (*p* < 0.05).

Abbreviations: CPA, cardiopulmonary arrest; ER, emergency room; ICU, intensive care unit; OR, odds ratio.

^a^ Pre‐admission CPA was defined as cardiopulmonary arrest occurring immediately prior to ICU admission, with successful resuscitation leading to ICU admission.

## 5 Discussion

This study analysed factors related to paediatric ICU patients' vital signs, conditions and backgrounds at ICU admission that could predict the likelihood of discharge to home and the need for medical care at home. The findings indicate two key points: first, the likelihood of discharge to home was independently predicted by the following factors—postoperative ICU admission, lower heart rate at admission, the absence of CPA immediately before ICU admission and no mechanical ventilation within the first hour of ICU admission. Second, the length of ICU stay emerged as a significant factor influencing the need for medical care after discharge.

Among the scoring systems predicting mortality outcomes in paediatric patients admitted to the ICU, the PIM 2/3 score includes variables such as ‘recovery from surgery or a procedure is the main reason for ICU admission’, ‘high‐risk diagnosis: cardiac arrest preceding ICU admission’ and ‘mechanical ventilation at any time during the first hour in ICU’ [4, 5]. These factors were also found to be significant in this study as determinants influencing the feasibility of home discharge, as demonstrated by the results of the multivariable analysis.

In infants, a smaller heart size and less myocardial contractility limit stroke volume, making an increased heart rate essential for maintaining cardiac output. As children grow, myocardial strength improves and stroke volume increases, resulting in a lower baseline heart rate [17]. Additionally, paediatric heart rate is more susceptible to fluctuations due to daily activities and other factors [18]. Severity assessment systems specific to paediatric populations, such as the Paediatric Early Warning Score (PEWS), incorporate heart rate as one of their criteria [19]. However, PEWS and other paediatric scores were primarily developed for the early detection of clinical deterioration, not for predicting discharge outcomes. In the original PIM, pulse rate was analysed but was not a significant predictor of mortality in univariate analysis [20]. In contrast, our study used home discharge rather than mortality as the outcome, and heart rate at admission remained an independent predictor even in multivariate analysis after adjusting for age. This discrepancy likely reflects the different study endpoints (mortality in PIM vs. feasibility of home discharge in our cohort). Nevertheless, our results indicate that heart rate at ICU admission, even after adjusting for age, was an independent predictor of the likelihood of discharge to home. In our cohort, a heart rate threshold of 131 bpm was identified as an indicator for possible discharge to home. However, the effect size of heart rate was relatively small, and the odds ratio was close to 1.0. Therefore, although the association was statistically significant, the clinical significance of heart rate as a predictor should be interpreted with caution. It may be more reasonable to consider heart rate in combination with other clinical factors rather than as a stand‐alone predictor.

As shown in Figure 2, although infants displayed a higher likelihood of not being discharged to home, no statistically significant relationship was observed solely through age stratification (data not shown). However, a higher heart rate among preschoolers and school‐aged patients at admission was associated with cases where discharge to home was not feasible, indicating that heart rate at ICU admission is significantly associated with discharge outcomes.

Previous studies have demonstrated a correlation between PIM3 scores and length of PICU stay, and high PIM2 scores at admission have been linked to longer PICU stays [8, 14]. While these studies do not focus specifically on the need for in‐home medical care, the similarity to our findings suggests that paediatric patients with prolonged ICU stays may also require additional medical care after discharge. Early intervention for discharge planning, particularly for patients expected to need extended in‐home care, may benefit these patients and their families.

Although ICU length of stay is not an admission variable and therefore cannot serve as a primary predictor, we considered it in a secondary analysis because prolonged ICU hospitalisation has practical implications for discharge planning and the early initiation of family education for home medical care.

## 6 Limitations

This study has several limitations. First, it was conducted retrospectively at a single university hospital, which may introduce selection bias towards more critically ill paediatric patients with complex medical needs and limit the generalisability of the findings. Second, the sample size was relatively small, which may have reduced the statistical power and widened confidence intervals. Third, outcomes for paediatric patients who were transferred to other hospitals could not be tracked, as follow‐up for such cases was practically unfeasible. Fourth, we did not calculate established severity‐of‐illness scores such as PIM2 or PIM3, as some required variables were not consistently available in the retrospective records. Therefore, further multicentre prospective studies with larger sample sizes and incorporating standardised severity scores are needed to validate and extend these findings. Nonetheless, these limitations do not appear to impact the present study's primary conclusions.

## 7 Implications for Practice

Early identification of paediatric ICU patients at higher risk of requiring medical care at home—using routinely available admission characteristics such as postoperative status, heart rate and the need for early mechanical ventilation—may enable healthcare professionals to initiate targeted discharge planning and family education at an early stage of ICU admission. In particular, recognising patients unlikely to be discharged home without ongoing medical support may facilitate timely coordination with multidisciplinary teams and community‐based services, thereby supporting a smoother transition to home care. In addition, prolonged ICU length of stay may serve as a practical signal to intensify preparation for post‐discharge medical support, including caregiver training and resource allocation.

## 8 Conclusion

This study investigated the associations between factors related to the condition and background of paediatric patients under the age of 16 at ICU admission, and the feasibility of discharge to home as well as the need for medical care at home. This study suggests that early and appropriate nursing interventions can be considered for paediatric ICU patients and their families, leading to the provision of high‐quality nursing care.

## Funding

The authors have nothing to report.

## Ethics Statement

This study was conducted with the approval of the Ethics Review Committee of Shiga University of Medical Science (approval number: R2023‐005, 14 April 2023). The study was registered in the University Hospital Medical Information Network (UMIN) Clinical Trials Registry (UMIN000050902).

## Consent

An opt‐out document was prepared, and consent to participate in the study was assumed based on its publication on the Shiga University of Medical Science website. The opt‐out document stated that it was possible to stop the use and provision of data in this study and clearly provided the opportunity to decline participation.

## Conflicts of Interest

The authors declare no conflicts of interest.

## Data Availability

The data that support the findings of this study are available from the corresponding author upon reasonable request.
